# The incidence of sore throat and group A streptococcal pharyngitis in children at high risk of developing acute rheumatic fever: A systematic review and meta-analysis

**DOI:** 10.1371/journal.pone.0242107

**Published:** 2020-11-18

**Authors:** Sarah Pearce, Asha C. Bowen, Mark E. Engel, Maya de la Lande, Dylan D. Barth

**Affiliations:** 1 Faculty of Health and Medical Sciences, University of Western Australia, Perth, Western Australia, Australia; 2 Wesfarmers Centre for Vaccines and Infectious Diseases, Telethon Kids Institute, Nedlands, Western Australia, Australia; 3 Department of Infectious Diseases, Perth Children’s Hospital, Nedlands, Western Australia, Australia; 4 Menzies School of Health Research, Charles Darwin University, Darwin, Northern Territory, Australia; 5 Institute for Health Research, University of Notre Dame, Fremantle, Western Australia, Australia; 6 Department of Medicine, University of Cape Town, Cape Town, Western Cape, South Africa; University of Auckland, NEW ZEALAND

## Abstract

**Background:**

Group A streptococcal (GAS) pharyngitis has traditionally been considered the sole precursor of acute rheumatic fever (ARF). Evidence from Australia, however, suggests that GAS skin infections may contribute to the pathogenesis of ARF. A missing piece of evidence is the incidence of sore throat and GAS pharyngitis in this setting. We conducted a systematic review and meta-analysis of the incidence of sore throat and GAS pharyngitis in all children at risk of developing ARF.

**Methods:**

Databases were systematically searched for studies reporting on the incidence of pharyngitis among children from low to upper-middle income countries, and Indigenous children living in high-income countries. Studies were subjected to data extraction by two independent reviewers. Following an assessment of the methodological quality of the studies, we extracted incidence rates (IRs) and conducted a meta-analysis. This systematic review is registered on PROSPERO (CRD42019113019).

**Results:**

From 607 titles identified by the search, 11 articles met the predetermined inclusion criteria; ten studies reported IRs while for the remaining study, the incidence was calculated. The pooled incidence estimated for sore throat was 82.5 per 100 child-years (95% confidence interval [CI], 6.5 to 1044.4 per 100 child-years, I^2^ = 100%) and GAS pharyngitis was 10.8 per 100 child-years (95% CI, 2.3 to 50.0 per 100 child-years, I^2^ = 99.9%).

**Conclusions:**

The pooled IRs for sore throat in children at risk of developing ARF were higher than rates reported in developed nations (32.70–40 per 100 child-years) and similar for GAS pharyngitis (12.8–14 per 100 years). The limited Australian data lend support to the need for further studies to inform the role of GAS pharyngitis in the development of ARF in Australian Indigenous children, so as to inform local primary prevention strategies for ARF and Rheumatic Heart Disease (RHD).

## Introduction

Acute pharyngitis is a common presentation to general practitioners [1]. Most cases of sore throat are caused by viruses; however, 25–30% of these infections are caused by the Group A Streptococcal (GAS) bacteria [2]. While sore throat is a minor illness, 0.3–3% of patients with GAS pharyngitis may develop serious complications, [2] such as acute rheumatic fever (ARF) and its sequela, rheumatic heart disease (RHD) [3]. ARF and RHD are a significant cause of morbidity and mortality in developing countries and amongst Indigenous populations living in developed countries [4]. In Australia, the incidence of ARF in Indigenous and Torres Strait Islander people is >300/1000 person-years, 60-fold greater than in non-Indigenous Australians (<5/1000 person-years).[5]

Traditionally, GAS pharyngitis is thought to be the sole precipitant of ARF; thus ARF primary prevention strategies have focused on treating GAS pharyngitis [6]. In Australian Indigenous communities, where ARF is endemic, GAS pharyngitis is seemingly rare [7], while the burden of GAS impetigo is amongst the highest reported in the world [8]. As such, impetigo has been proposed as an additional driving force behind ARF in these communities. If impetigo is established to be a ‘bona fide’ precipitant of ARF, there would be major implications for primary prevention strategies, which are currently directed primarily towards the treatment of GAS pharyngitis [2]. The shift towards including GAS impetigo, (a condition often normalized or neglected by health care providers as a benign condition [9]) in RHD prevention strategies has already begun with an emphasis on healthy skin in the third edition of the 2020 guidelines for the prevention and management of RHD in Australia (https://www.rhdaustralia.org.au).

The evidence for the skin-ARF theory is based mostly on epidemiological studies and observations from Northern Australia [6]. Impetigo is highly prevalent in this region, affecting a median of 45% of Indigenous children at any point in time [8]. Molecular epidemiological studies also show that the pharyngeal strains usually responsible for ARF (rheumatogenic strains) are absent, and there is significant overlap between the typically distinct skin and throat strains [6]. The notion that sore throat is uncommon is largely anecdotal and informed by reports from Indigenous Health Workers, nurses, and medical officers working in Northern Australia. Furthermore, there is a paucity of published data on the frequency of Indigenous children being treated for GAS pharyngitis in remote regions. Only two published studies that report the incidence of sore throat in this setting, and they yield conflicting results [7, 10]. Given that the skin-ARF hypothesis hinges on the idea that sore throat is uncommon, the lack of evidence is surprising and must be addressed. A large, longitudinal, prospective cohort study of GAS pharyngitis and impetigo with episodes of ARF as the primary endpoint would be ideal, but time, cost and remoteness of the large, sparsely populated region preclude this.

To address this question from a different angle, we have systematically compiled the available evidence on the incidence of sore throat and GAS pharyngitis in other populations where ARF is endemic, to compare and contrast with known incidence for Australian Indigenous children. Incidence has been chosen, rather than prevalence, as it provides data on the number and frequency of new episodes of pharyngitis, and over time, the increasing risk of ARF from recurrent episodes [11, 12].

## Methods

This systematic review and meta-analysis was conducted in accordance with the Preferred Reporting Items for Systematic reviews and Meta-Analysis (PRISMA) guidelines (S1 Checklist) [13] and is registered on PROSPERO (International Prospective Register of systematic reviews, registration number CRD42019113019, https://www.crd.york.ac.uk/PROSPERO).

### Review question

This review asks the following questions. What is the incidence of sore throat and GAS pharyngitis in children at high risk of ARF? Is there variation in incidence with respect to geography, climate, health care setting or home setting?

### Search strategy and data sources

We reviewed similar systematic reviews reporting on the epidemiology of pharyngitis and skin infections in children from low income countries or Indigenous children living in high-income areas [8, 14], in order to construct the search terms and avoid overlap with available evidence. Following this, the search strategy was developed to capture all relevant studies, using Medical Subject Headings (MeSH) combined with key words (Table 1). PubMed, Ovid Medline, and Embase databases were searched, then supplemented with grey literature from searches of the Australian Institute of Health and Welfare (AIHW), Australian Indigenous HealthInfoNet, Informit, Agency for Healthcare Research and Quality (AHRQ), and The Grey Literature Report databases. To identify as many studies as possible, no filters were applied to limit age, language, publication date or study design. Results were also supplemented by searching the references of the selected papers and their citations through Web of Science. An update on the search was conducted in PubMed on 11^th^ July 2020.

**Table 1:** Search strategy.

Incidence [MeSH term] OR burden OR epidemiology OR rate
AND
Pharyngitis [MeSH term] “sore throat” OR pharyngitis OR GAS OR Group A Streptococc* OR streptococc* OR streptococc* pyogenes OR streptococc* pyogenes pharyngitis OR group A streptococcal pharyngitis OR tonsillitis OR tonsillopharyngitis OR streptococcus pyogenes [MeSH Terms]
AND
Indigenous OR aborigin* OR torres strait islander OR oceanic ancestry group OR maori OR pacific islander OR native American OR American Indian OR inuit OR minority group OR first nation OR native people OR developing countries OR developing nation OR low income countries OR low income nations OR low-middle income countries OR low-middle income nations OR middle income countries OR middle income nations OR less developed countries OR less developed nations OR third world countries OR third world nations OR poverty OR socioeconomic inequality OR living standards OR resource limited OR low resource

MeSH, Medical subject heading

### Study selection

Inclusion criteria

1. Papers reporting the incidence of pharyngitis and/or studies with a follow-up period from which incidence could be calculated
2. The population under investigation was children from low- to middle-income countries or Indigenous children living in high-income countries
3. GAS diagnosed based on culture and/or a rapid antigen detection test

Exclusion criteria

1. Prevalence studies of pharyngitis
2. Molecular typing studies
3. Any studies lacking primary data including narrative reviews, opinion pieces, or letters
4. Duplicate publications of the same data; where this occurred the most recent data were used

### Case definitions

The method of case ascertainment for all studies involved a clinical assessment, and a pharyngeal swab, and microbiological culture for GAS bacteria. Symptomatic sore throat was defined simply as a participant complaining of throat pain [15]. GAS pharyngitis was defined as a child complaining of a sore throat, with confirmation of GAS detection in the pharynx. GAS detection was either culture confirmation of *Streptococcus pyogenes* from a throat swab collected and processed in the laboratory or detection of the presence of the *S*. *pyogenes* antigen using a rapid antigen detection test. Whilst culture confirmation is the gold standard, RADT kits are used in many jurisdictions for prompt detection of *S*. *pyogenes* at the point of clinical care and have a reported sensitivity of between 80 and 95% [16].

### Study records

#### Selection process

All included titles were compiled, and duplicates removed. Two reviewers (SP & MD) independently screened the references for eligibility using a numbered coding system to record the decision for each article. At each step of the process reviewers would meet and compare their decisions; discrepancies were discussed and where necessary a third reviewer (DB) resolved any disagreements. The titles and abstracts were screened for relevance, and potential full-text articles downloaded. The full-text articles were reviewed in depth and the final articles were then selected for inclusion.

#### Data extraction

The two reviewers independently extracted data from the included papers and entered them into a data extraction form using three Excel *pro forma*. The extracted data were categorised into general information, study characteristics, and incidence calculations. General information included the study ID, authors, year of publication, aim of study, start date, end date, and season of participation. The study characteristics included region, country, climate, income, study setting, geographical setting (rural/urban/peri-urban), population description, inclusion criteria, exclusion criteria, site of recruitment, selection methods, age range, ethnicity, pharyngitis definition, and method of ascertainment. Climate was determined by the Köppen-Geiger Climate Classification system [17] and country income by the World Bank income classification (at the time the study was conducted). To calculate the IR for sore throat and GAS pharyngitis, we extracted data on the population denominator (N), number of new cases (n), incidence density per 100 child-years, and the confidence intervals (CI). Where sample populations included both symptomatic and asymptomatic pharyngitis, we only extracted information from symptomatic participants [18]. Incidence was defined as the number of new cases (n) over the study period for each child (N) as per the formula below [19].

$$Incidence\;per\;100\;child-years=\frac{Number\;of\;new\;cases\;during\;period(n)}{\left(Number\;of\;participants\times days\;of\;observation)(N\right)}\times 365\times 100$$

The included studies used a variety of methods to report incidence. As such, using the available data, we recalculated all reported incidences using this formula to adjust for studies where incidence was not reported or where different units were used, e.g. 1000 child-weeks [7, 10].

#### Quality assessment

Limited tools are available to assess the methodological quality of observational studies [20]. A tool developed for prevalence studies by Hoy et al. [21], and adapted by Werfalli et al. [22], was modified for our prevalence review. Question nine—*‘Was the length of the shortest prevalence period for the parameter of interest appropriate*?*’* was changed to ‘*Was the follow-up period for the parameter of interest appropriate*?*’* The two independent reviewers used the tool to score the studies as high risk (score<6), moderate risk (6–8) and low risk (>8) of bias.

#### Statistical analysis

We calculated pooled incidence estimates for both sore throat and GAS pharyngitis from studies with available incidence or where we could calculate incidence using the formula above. Meta-analysis models were applied by using the log IRs of sore throat and GAS pharyngitis and corresponding standard errors (metan routine: metan logirr sologirr), using Stata^®^ version 16. The random effects model was used, and heterogeneity was quantified using the I^2^ statistic. Sensitivity analysis was performed to explain the potential sources of heterogeneity. The analysis included plotting the pooled IR of studies by sample size. Subgroup analyses were performed to compare pooled estimates by country, geographical setting, study setting, climate, and income status according the World Bank Index at the time the studies were conducted. We were unable to conduct a funnel plot analysis to examine for publication bias given too few studies included in our mete-analysis. Where meta-analysis was not possible due to a lack of data or differences in the methods, a narrative review of the results is provided.

## Results

### Literature search

**Fig 1:**
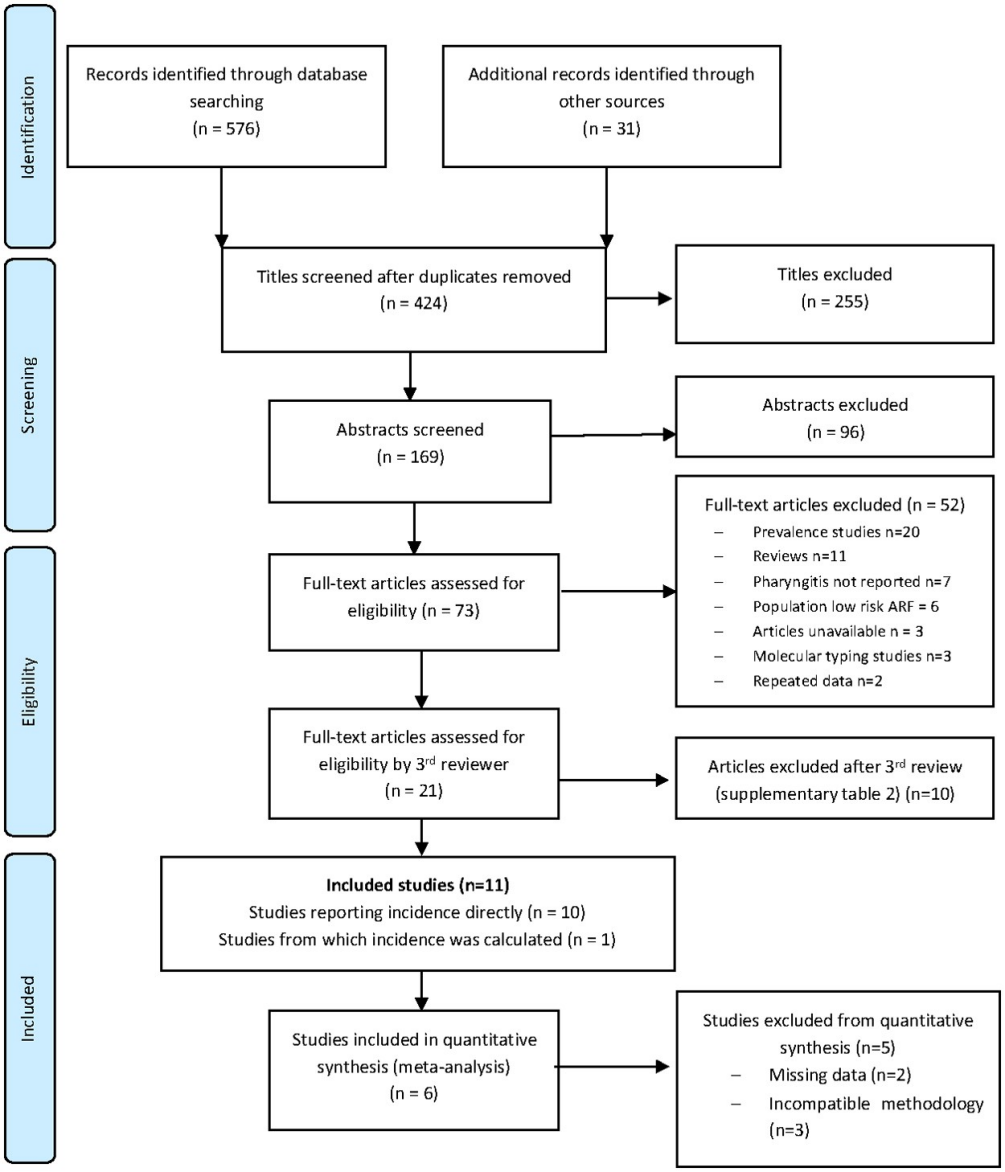
PRISMA flowchart.

Between October 2018 and October 2019, 607 titles were identified by the search strategy (Fig 1). Duplicate removal rendered 424 titles for assessment by two reviewers (SP and MD); a third reviewer (DB) adjudicated where needed. Twenty-one studies were deemed potentially eligible, from which 11 were included after a second review by DB. Ten studies directly reported the IRs [7, 10, 15, 18, 23–28] and we calculated the incidence from a single study [29]. Excluded studies are reported in S1 Table.

### Study characteristics

Data from more than 16,814 participants over 46-years were included from the eleven studies (Table 2) [7, 10, 15, 18, 23–29]. Studies were conducted in eight countries, predominantly from Asia or Oceania, in a range of income settings. The climate ranged from arid to tropical, with a mixture of rural and urban households. The studies were conducted in either a school or health care clinic setting. A summary of the IRs, country income level and climate of included studies are documented in Table 3.

**Table 2:** The characteristics of included studies.

Study ID	Country	Age	Sample Size (children)	Study Design	Site of recruitment	Sampling Method	Inclusion criteria	Ethnicity	Urban/Rural	Method of Ascertainment
**El-Kholy 1972** [18]	Egypt	6–12	156	Prospective Cohort Study	School	Convenience	Any child attending the primary school on a regular basis, present on the days of assessment.	Egyptian	Rural	Weekly clinical assessment, monthly GAS pharyngeal swabs, and 6-monthly ASOT. No clinical observations of pharyngitis made so monthly ASOT were implemented.
**Nicolle 1990** [24]	Canada	4–14	Rankin Inlet: 309 St. Therese Point: 449	Prospective Cohort Study	School	Convenience	Children regularly attending primary school and children presenting to the nursing station with pharyngitis or impetigo	Inuit, Caucasian & Native Indian	Rural	3x weekly visits and presentations to nursing station: clinical assessment; swab + culture if symptomatic
**Lennon 2000** [26]	New Zealand	5–15	Not specified	Prospective cohort study	School	Convenience	Children at one of the 25 high risk schools with consent from parents.	Not specified but includes Maori/Pacific Islander	Urban	Daily swabs by lay workers (mostly parents). Any child with a positive culture was referred for clinical assessment and treatment.
**Nandi 2001** [25]	India	5–15	536	Prospective Cohort Study	Household	Quasi-randomisation	School aged children living in Chandigarh chosen at random.	Indian	Periurban	Fortnightly clinical assessment; swab + culture if symptomatic
**McDonald 2006** [7]	Australia	0–15	1173 adults + children (number of children not specified)	Prospective Cohort Study	Households with known history of RHD	Convenience	Any person living in a household with a known history of ARF and/or RHD.	Indigenous Australian	Rural	Monthly clinical assessment, swab, culture + ASOT
**McDonald 2007** [10]	Australia	0–15	91	Prospective Cohort Study	Households with known history of RHD	Convenience	Any person living in a household with a known history of ARF and/or RHD.	Indigenous Australia	Rural	Monthly clinical assessment, swab, culture + ASOT
**Steer 2009** [15]	Fiji	7–12	685	Prospective Cohort Study	School	Convenience	Children between 5–14 years old located in one of four schools chosen for the study.	Indigenous Fijian Indo-Fijian Other	2 rural, 2 urban	Fortnightly clinical assessment; swab + culture if symptomatic
			Urban = 426							
			Rural = 241							
**Kumar 2009** [29]	India	5–15	4249	Prospective Cohort Study	School	Quasi-randomisation	Children aged 5–15 attending school, with parental consent	Indian	Rural	Fortnightly clinical assessment, swab + culture
**Engel 2012** [28]	South Africa	5–15	950	Prospective study	Clinic	Convenience	Children aged 5–15 years presenting to the clinic with pharyngitis	Black African Mixed ancestry	Periurban	Daily clinical assessments, swabs and culture
**Kumar 2012** [27]	India	7–11	241	Prospective Cohort Study	School	Convenience	Children attending village primary schools	Indian	Rural	Fortnightly clinical assessment, swab + culture
**Wu 2016** [23]	China	0–14	Not specified	Multiplier model based on paediatric outpatient clinic	Paediatric outpatient clinics	Convenience	Children aged 0–14 years diagnosed with pharyngitis or scarlet fever.	Chinese	Urban	Clinical assessment, swab + culture. Multiplier model used to calculate incidence.

ASOT, Anti-Streptolysin A Titre; NS, Not Stated; ARF, Acute Rheumatic Fever; RHD, Rheumatic Heart Disease

**Table 3:** Summary of incidence of sore throat and GAS pharyngitis in children at risk of acute rheumatic fever.

Study ID	Pharyngitis Incidence (per 100 child-years)	GAS Pharyngitis Incidence (per 100 child-years)	Country, Region	Climate	Income
**El-Kholy 1972** [18]	Not done	31.2	Egypt, Africa	Arid	Low-middle
**Nicolle 1990** [24]	Not done	Rankin Inlet: 4.9 St Therese Point: 9.4	Canada, North America	Cold	High
**Lennon 2000** [26]	Not done	50	New Zealand	Temperate	High
**Nandi 2001** [25]	705	95	India, Asia	Temperate	Low
**McDonald 2006** [7]	Adults + children: 19	Adults + Children: 4	Australia, Oceania	Tropical	High
	Children: 8	Children: 0			
**McDonald 2007** [10]	Adults + children: 480	Adults + Children: 32	Australia, Oceania	Arid	High
	Children: 457	Children: Not done			
**Steer 2009** [15]	162.9	14.7	Fiji, Oceania	Tropical	Upper-middle
**Kumar 2009** [29]	232	5.4	India, Asia	Temperate	Low
**Engel 2012** [28]	0.84	0.18	South Africa	Temperate	Upper-middle
**Kumar 2012** [27]	Not done	16.6	India, Asia	Temperate	Low
**Wu 2016** [23]	29.8	2.68	China, Asia	Cold	Upper-middle

GAS, group A streptococcus

Study designs were predominantly prospective cohort studies which followed children, either through school, household or clinic visits (Table 2) [7, 10, 15, 18, 23–29]. A single study used a multiplier model based on surveillance of sore throat and GAS pharyngitis through 36 pediatric outpatient clinics to calculate incidence [23]. Most studies used convenience sampling except Lennon 2000 [26], Nandi 2001 [25], and Kumar 2009 [29], which each used quasi-randomisation. Most studies swabbed all children [7, 10, 18, 23, 26, 27, 29] while others only swabbed those who were symptomatic [15, 24, 25, 28]. Two studies collected venous blood samples for anti-streptolysin O titres (ASOT) and used an alternative incidence calculation: incidence = ST/OB/3.5 x 365 x 100 (ST = sore throats, OB = number of observations, 3.5 average duration of sore throat in days) and did not report enough data to allow recalculation [7, 10]. El Kholy 1972 defined GAS pharyngitis as a child who had a positive GAS pharyngeal culture two weeks prior to a rise in the ASOT, because there were no clinical cases of pharyngitis [18].

### Meta-analysis

#### Incidence of sore throat and GAS pharyngitis

**Fig 2:**
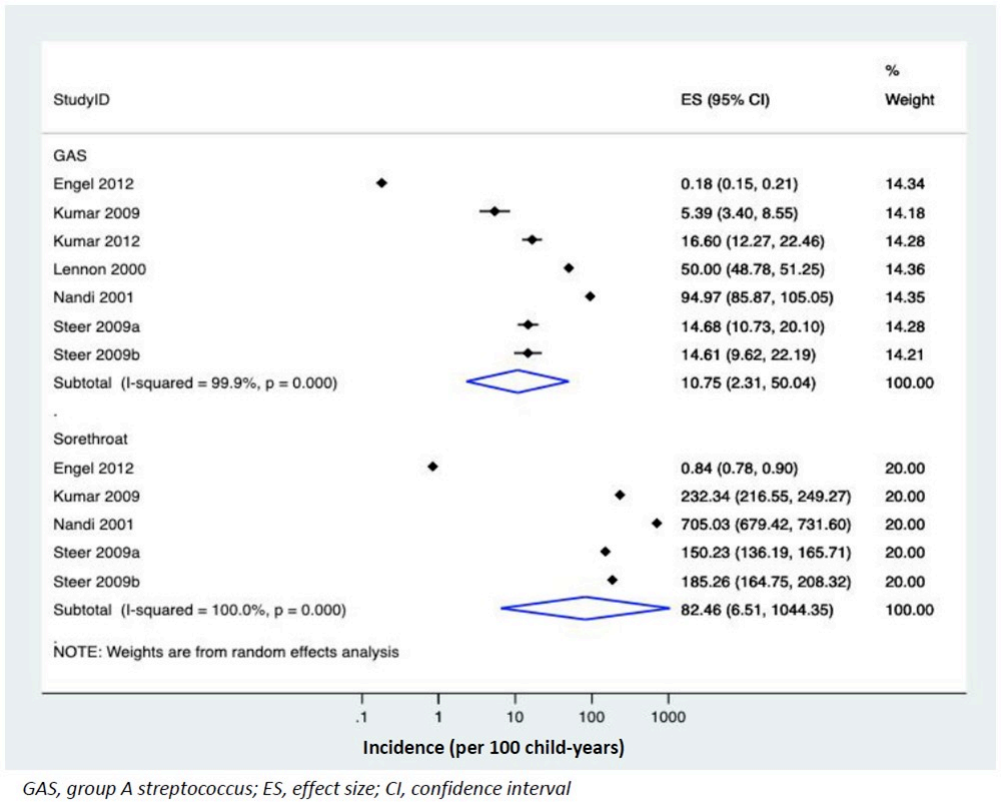
Pooled incidence rates of GAS pharyngitis and sore throat in children at risk of acute rheumatic fever.

The pooled IRs in children aged 5–15 years for sore throats was 82.5 per 100 child-years (95% CI, 6.5 to 1044.4; 4 Studies, n = 6,420; I^2^ = 100%) [15, 25, 28, 29] and GAS pharyngitis was 10.6 per 100 child-years (95% CI, 2.3 to 50.0; 6 studies, n = 6,661, I^2^ = 99.9%) [15, 25–29] (Fig 2).

#### Subgroup analyses

**Fig 3:**
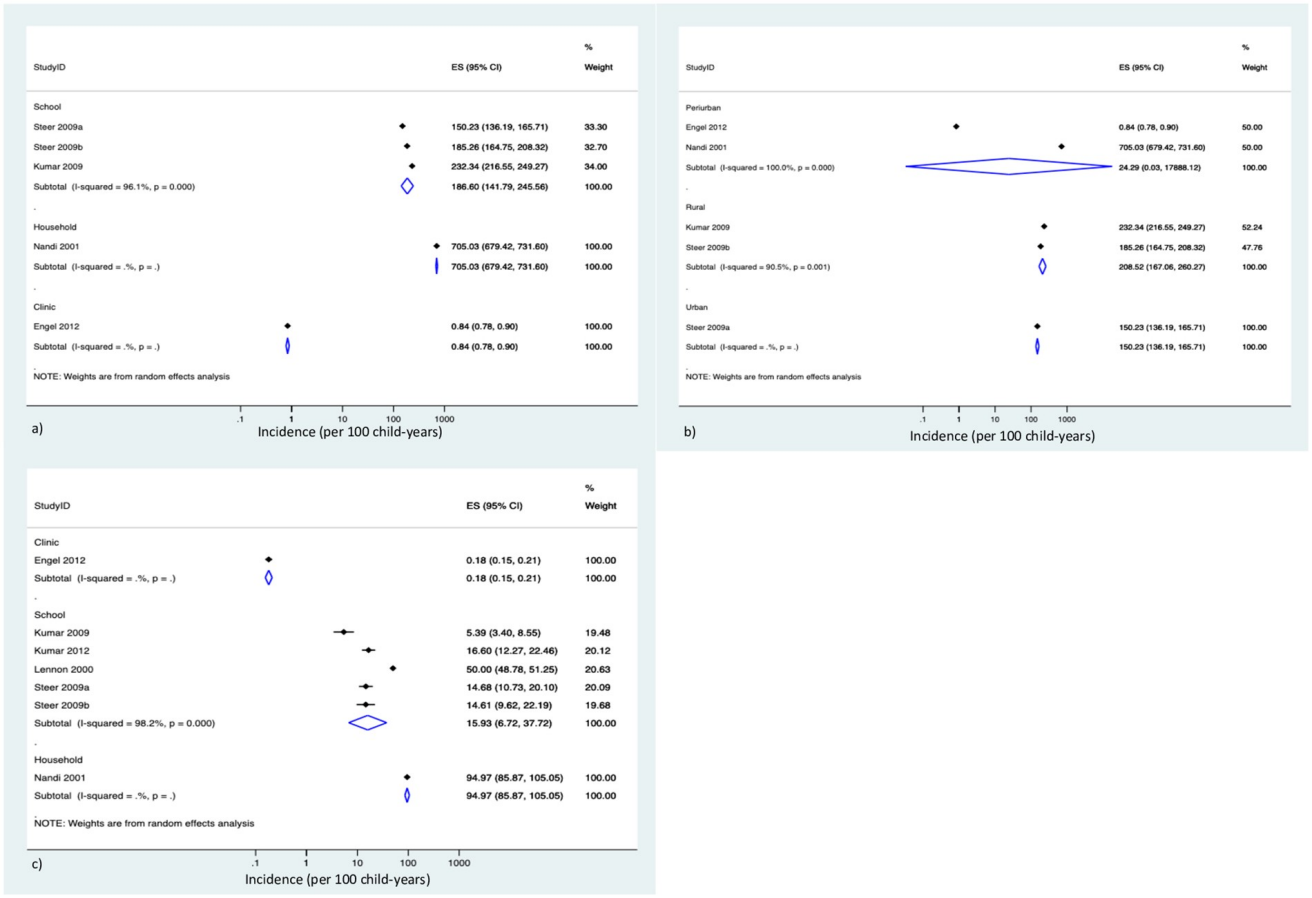
Subgroup analysis of pooled incidence rates: 3a) Sore throat IRs by study setting, 3b) sore throat IRs by rurality, and 3c) GAS pharyngitis by study setting. ES, effect size; CI, confidence interval.

We further conducted analyses in each of the sore throat and GAS pharyngitis subgroups, with respect to various parameters. No significant difference in sore throat incidence was observed according to climate: temperate IR = 51.6 per 100 child-years (95% CI, 1.1–2529.7; 3 studies, n = 1710) [25, 28, 29] vs tropical IR = 166.4 per 100 child-years (95% CI, 135.5–204.3; 1 study, n = 685) [15]. Studies conducted in schools had a sore throat IR of 186.6 per 100 child-years (95% CI, 141.8 to 245.6; 2 studies, n = 1019) [15, 29] compared with 705.0 per 100 child-years (95% CI, 679.4 to 731.6; 1 study, n = 536) and 0.8 per 100 child-years (95% CI, 0.8 to 0.9; 1 study, n = 840) in a single study reporting household [25] and clinic [28] settings, respectively (Fig 3a). Studies conducted in rural settings had a significantly higher pooled IR of sore throat 208.5 per 100 child-years (95% CI, 167.1 to 260.3; 2 studies, n = 613) [15, 29] compared to those conducted in urban settings of 150.2 per 100 child-years (95% CI, 136.2 to 165.71; 1 study, n = 399) per 100 child-years [15] (P<0.05). The pooled IR in peri-urban settings were found to be 24.3 per 100 child-years (95% CI, 0.03 to 17888.1; 2 studies, n = 1376) [25, 28] (Fig 3b).

For GAS pharyngitis, schools had a pooled IR of 15.9 per 100 child-years (95% CI, 6.7 to 37.7; 4 studies, 13,260 participants) [15, 26, 27, 29]. This IR was significantly higher than that in the single study conducted in a clinic setting (IR = 0.2 per 100 child-years (95% CI, 0.2 to 0.2; 1 study, n = 840)) [28] and significantly lower than the IR in studies conducted in in household settings (IR = 95.0 per 100 child-years (95% CI, 85.9 to 105.1; 1 study, n = 536) [25] (Fig 3c).

By country, New Zealand had the highest pooled IR of 50 per 100 child-years (95% CI, 48.8 to 51.3; 1 study, n = 12 000) [26] followed by India, 20.6 (95% CI, 3.8 to 112.8; 3 studies, n = 1111) [25, 27, 29] and Fiji, 14.7 (95% CI, 11.4 to 18.8; 1 study, n = 685) [15]. South Africa had a significantly lower incidence compared with the other regions with an IR of 0.2 per 100 child-years (95% CI, 0.2 to 0.2; 1 study, n = 840) [28] (P< 0.05).

### Narrative summary of studies not included in the meta-analysis

Additional studies, though not amenable to meta-analysis, provide additional data on the incidence of sore throat [7, 10, 18, 23, 24, 26, 27] and GAS pharyngitis [7, 10, 18, 23, 24]. The Australian and Canadian studies recruited Indigenous children from high-income countries, the Chinese study, children from the upper-middle income bracket [30, 31], while the Egyptian study represents a low-to-middle income country. The studies were prospective cohort studies conducted amongst households [7, 10] and schools [24]. The remaining study used a multiplier model to estimate the incidence based on presentation to pediatric outpatient clinics [23]. (Table 2).

The incidence of sore throat ranged from 8–457 per 100 child-years (3 studies, n = >1264) [7, 10, 23]. The highest incidence observed was in arid, Central Australia, whereas the lowest incidence was found in tropical, Northern Australia. The study from China fell in-between this range. For GAS pharyngitis, the IRs ranged from 0–32 per 100 child-years (5 studies, n = >2178) [7, 10, 18, 23, 24] Similarly, the highest incidence occurred in arid, Central Australia and the lowest in tropical, Northern Australia, in which no cases were observed.

In Australian Indigenous children, the incidence of sore throat was reported to be 8 and 457 per 100 child-years in Northern [7] and Central Australia [10] respectively. The incidence of GAS pharyngitis was 0 per 100 child-years in Northern Australian children [7], and 32 per 100 person-years in Central Australian adults and children (data for children alone was not reported) [10].

### Sensitivity analysis

Given the large heterogeneity, I^2^ = 100% and I^2^ = 99.9% for sore throat and GAS pharyngitis respectively, we assessed the influence of sample size which showed no statistical difference for studies considered to be adequately powered compared with those inadequately powered (P>0.05). Country income level according to the world bank classification at the time the study was conducted was also considered and there was no statistical difference in rates by income category. We intended to pool the IRs by risk of bias assessment, however, all the included studies in the meta-analysis had a low risk of bias.

#### Assessment of risk of bias in included studies

Eight studies had a low risk of bias, [7, 10, 15, 25–29] and three had a moderate risk of bias, [18, 23, 24] (S2 Table; S1 Fig).

## Discussion

This systematic review is the first comprehensive synthesis of the incidence of sore throat and GAS pharyngitis in children at risk of ARF. The main findings are:

1. The pooled incidence of sore throat in children at risk of ARF is 82.5 (95% CI, 6.5 to 1044.4) per 100 child-years.
2. The pooled incidence of GAS pharyngitis in children at risk of ARF is 10.8 (95% CI, 2.3 to 50.0) per 100 child-years.

The pooled IR for sore throat (82.5 per 100 child-years) is higher than estimates reported elsewhere [32]. A prospective surveillance study conducted in Melbourne reported IRs for sore throat ranging from 32.70–40 per 100 child-years (urban children, ages 0–18 years) [32]. In an urban setting at lower risk of ARF, the incidence of sore throat was far lower than what was found in our study [33]. The pooled incidence of GAS pharyngitis; however, was comparable to other studies. A prospective surveillance study of GAS pharyngitis in former Czechoslovakia in 1984 reported an incidence of 7.2 per 100 person-years (urban children and adults) [34]. Two studies in urban families in Melbourne, Australia were also conducted in the early 2000s. The first study comprising children and adults found an IR of 14 per 100 person-years for GAS pharyngitis [32]. The second study reported IRs for GAS pharyngitis ranging from 9.2–12.8 per 100 child-years in children aged 0–18 years [33]. Interestingly, a study conducted in Philadelphia, USA in the 1950s reported the IR for GAS pharyngitis of 15.5 per 100 child-years, [18] however, some of the included schools were part of a penicillin treatment program implying that their findings could be an underestimate.

Our meta-analysis also highlights that there is a stark difference between the incidence of sore throat and GAS pharyngitis, almost 8-fold lower (82.5 versus 10.8 per 100 child-years, p<0.05). A recent prevalence study; however, reports that the proportion of GAS causing pharyngitis is between 20 and 30% [3]. Therefore, one might expect that we would have found approximately a third the rate of GAS pharyngitis compared to sore throats. The 10-fold lower rate may be improved in precision with more studies to accurately describe the incidence of GAS pharyngitis.

We considered the impact of study setting, geographical setting, country, climate and country income level on the overall incidence estimates of sore throat and GAS pharyngitis in children at risk of developing ARF. The IR for both sore throat and GAS pharyngitis was lowest in studies conducted in the clinic setting and highest in studies conducted in households. These differences are to be expected; clinic-based studies are likely to see more severe disease rather than the mild cases that would not warrant a visit to the clinic. The high incidence in the household setting may be explained by socio-economic risk factors including overcrowding, given that the study was undertaken in Indian Slums and would therefore be surveying both mild and severe disease. The school setting is a convenient site for recruitment given the population of interest and children with sore throat may not always warrant a visit to the clinic. Another strength of school-based surveillance is that even though a child with sore throat may be missed due to absenteeism, depending on how often surveillance takes place, it is possible to evaluate that case upon return if symptoms are still present. We also compared the pooled IR by each setting to the overall pooled IR for sore throat and GAS pharyngitis. In the school setting, the pooled IR for sore throat of 82.5 (95% CI, 6.5 to 1044.4) per 100 child-years was similar to overall pooled estimate of 186.6 (95% CI, 141.8 to 245.6) per 100 child-years. Similarly, for GAS pharyngitis, the overall pooled IR of 10.8 (95% CI, 2.3 to 50.0) per 100 child-years was similar to the pooled IR of 15.9 (95% CI, 6.7 to 37.7) per 100 child-years found in the school setting. This finding suggests that the school settings is probably a more accurate representation of the incidence among children at risk of ARF compared to studies conducted in clinic or household settings, which may either under or overestimate the true burden of disease in this population.

When we considered the impact of geographical setting, studies conducted in rural settings had a significantly higher IR compared with urban settings and may be explained by socioeconomic factors which influence infection such as household overcrowding, particularly in rural living indigenous communities. This difference is in contrast to findings in a systematic review on the prevalence of GAS pharyngitis conducted in Africa, where risk factors such as overcrowding are more likely to occur in urban settings [35].

A striking feature of this systematic review is the markedly higher IR of both sore throat and GAS pharyngitis reported by a study conducted in India [25] compared to the study conducted in South Africa [28]. While both studies were conducted in peri-urban communities of low socio-economic status, South Africa is considered to be an upper-middle income country with better living conditions compared with India as a low-income country. Another reason for this disparity could be the recruitment setting in which these studies were conducted. We also observed a significant difference in IR’s between studies conducted in Northern India. Both studies by Kumar *et al*. were conducted in the villages of the Panchkula district with better socio-economic conditions compared to the Nandi *et al*. study conducted in the slums of Chandigarh.

An interesting observation among the studies not amenable to meta-analysis, was the contrast between the results of two studies conducted in remote Indigenous communities [7, 10]. Both were conducted in households with a history of RHD, yet the community in Central Australia [10] had markedly higher incidences of sore throat and GAS pharyngitis, while the Top End of the Northern Territory [7] community had extremely low rates of sore throat and GAS pharyngitis. Two distinct differences between the communities would be their climates, tropical compared to arid, and their socioeconomic status, with the Central Australian community noted to be better resourced and have less household crowding than the Top End community [10]. The difference in resources would be unlikely to account for the higher IRs in the Central Australian community, as one would expect rates to be lower with less household crowding [10]. The tropical climate could be an important factor contributing to the contrasting rates. The study by Steer 2009 was the only other study conducted in a tropical country, and the IRs of sore throat and GAS pharyngitis were higher than in Top End Northern Australia, in fact they were quite close to the IRs found in the Melbourne studies. Without other studies, it is impossible to infer that there is an effect of the tropical climate. Overall, there is no satisfactory explanation for the contrast between the results of the two McDonald studies, however, monthly surveillance visits were conducted and thus could have missed many episodes of sore throat and GAS pharyngitis, warranting a more robust methods to estimate the true burden in those settings.

A strength of this review was the broad inclusive search strategy to avoid missing appropriate studies. We also recalculated incidence from appropriate studies to ensure that the same IR were being compared. The risk of bias analysis also highlights a strength, in that all included studies had either a low or moderate risk of bias, none were at high risk. The robust meta-analysis method allowed a pooled incidence estimate to be calculated from appropriate studies. Limitations include difficulty in capturing the ‘children at risk of ARF’ in the search strategy, even with guidance from previous articles. It was difficult to differentiate viral pharyngitis with GAS pharyngeal colonization from true GAS pharyngitis, which may have affected the results; although this was a limitation across all studies and is an ongoing clinical challenge in understanding the microbiology of pharyngitis. There was also a variation in methods used to establish the incidence of GAS pharyngitis and convenience sampling may have influenced the incidence rates during a particular period of time. Most studies used a swab and culture to confirm GAS pharyngitis, though some also included serological testing, which is not the gold standard. The variability in methods may have effected results and the utility of comparison.

The findings of this study have implications for both clinical practice and research. For clinicians, these results indicate that children from low to upper-middle income countries or Indigenous children in high-income countries suffer a high burden of sore throat and GAS pharyngitis. Other GAS infections such as impetigo may also be an important risk factor for developing ARF and thus these children should be managed with the prevention of ARF in mind [36]. These results also reinforce the importance of primary prevention programs with the focus on treating GAS pharyngitis to prevent ARF. A number of questions remain unanswered, particularly the disparity between sore throat and GAS incidence in the two McDonald studies from Australia. More research is warranted in the Indigenous Australian population living in Central and Northern Australia where there are some of the highest reported rates of ARF in the world [5].

## Conclusion

Our results confirm the high incidence of sore throat and GAS pharyngitis infections in children at risk of developing ARF, warranting the need for improved prevention and treatment strategies especially in remote regions and in Indigenous populations. Despite being a common condition [1] and well evidenced to precede ARF [2], there are few available studies describing the incidence of sore throat or GAS pharyngitis in children globally. This understudied condition makes it difficult to use the available evidence to inform estimates of sore throat and GAS pharyngitis for Australian Indigenous children at high risk of ARF and thus emphasizes the need for more research in the context of a high burden of impetigo. Finally, our findings support the call to accelerate the development of a GAS vaccine as a primary prevention strategy to reduce the burden of GAS diseases in populations at risk of developing ARF [37].

## Supporting information

S1 Checklist Checklist of the preferred reporting items for systematic reviews and meta-analyses for the systematic review of sore throat and GAS pharyngitis in children at risk of acute rheumatic fever. (DOCX)

S1 Table List of excluded studies following full text review [38–46]. (PDF)

S2 Table Summary of risk of bias analysis for studies reporting the incidence of sore throat and GAS pharyngitis in children at risk of acute rheumatic fever. Quality score: 0–5 high risk of bias (pink), 6–8 moderate risk of bias (yellow), > 8 low risk of bias (green).
(PDF)

S1 Fig Risk of bias chart for studies reporting the incidence of sore throat and GAS pharyngitis in children at risk of acute rheumatic fever. Green corresponds to low risk of bias, yellow corresponds to moderate risk of bias.
(TIF)
