# Supplementary material for: The incidence of sore throat and group A streptococcal pharyngitis in children at high risk of developing acute rheumatic fever: A systematic review and meta-analysis
Source: PLoS One. 2020 Nov 18;15(11):e0242107. doi: 10.1371/journal.pone.0242107 (PMC7673496; doi:10.1371/journal.pone.0242107)
Supplement: S1 Table — (PDF) [file pone.0242107.s002.pdf]

Study ID	Year	Reason for Exclusion
Nicolle 1988 [38]	1988	Prevalence study only included pharyngeal carriage not pharyngitis. Appears to use the same data as Nicolle 1990
Sarkar 1988 [39]	1988	Three-point prevalence studies conducted over one year to analyse the change with season.
Rotta 1989 [34]	1989	Does not distinguish between children and adults
Nicolle 1990 [40]	1990	Reports prevalence of GAS pharyngitis and outcome of antibiotic therapy.
Tewodros 1992 [41]	1992	Prevalence study conducted through outpatient clinics
Majeed 1993 [42]	1993	High income country
Smeesters 2006 [43]	2006	Prevalence study, no follow up period
Tartof 2011 [44]	2011	Period prevalence, no follow up period for each child
Tapia 2015[45]	2015	Appropriate prospective surveillance study; however, doesn't report denominator of children surveyed so incidence cannot be calculated. We attempted to contact the authors to obtain this information but were not successful.
Shay 2017 [46]	2017	Reports incidence but calculated through a retrospective survey with no follow up period. Also, high income country with no Indigenous population specifically identified.
