# Supplementary figures and images for: The incidence of sore throat and group A streptococcal pharyngitis in children at high risk of developing acute rheumatic fever: A systematic review and meta-analysis
Source: PLoS One. 2020 Nov 18;15(11):e0242107. doi: 10.1371/journal.pone.0242107 (PMC7673496; doi:10.1371/journal.pone.0242107)

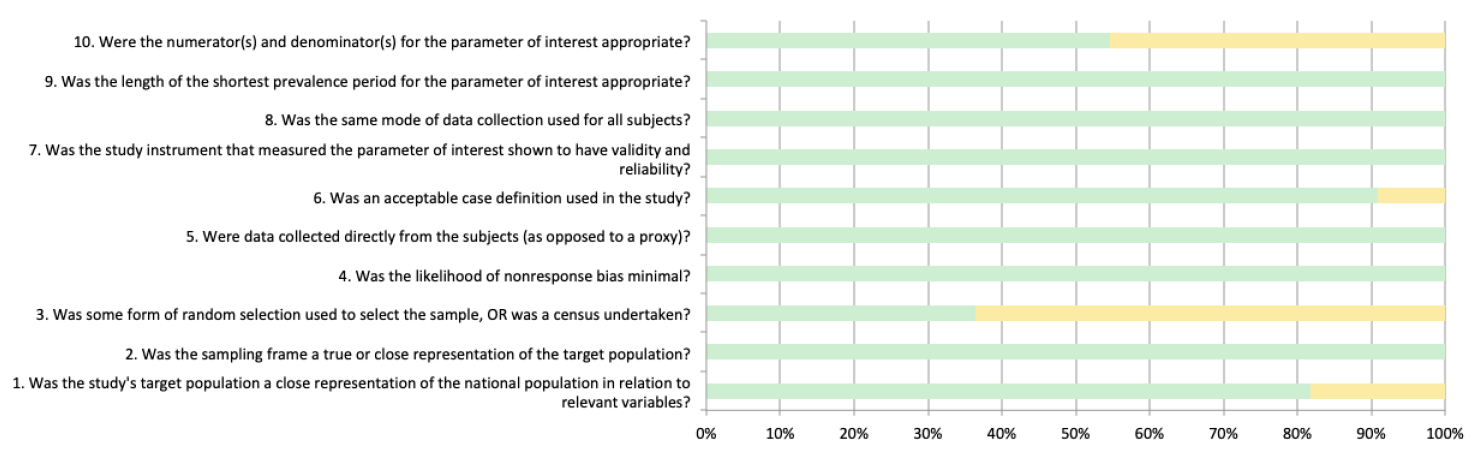

Supplement: S1 Fig — Green corresponds to low risk of bias, yellow corresponds to moderate risk of bias. (TIF) [file pone.0242107.s004.tif]
